# Significance of an endoscopically injected nodule detected on ultrasound as a predictive factor for the resolution of vesicoureteral reflux

**DOI:** 10.3892/etm.2015.2206

**Published:** 2015-01-22

**Authors:** DONG-GI LEE, SIN WOO LEE, KWAN HYUN PARK, DONG SOO RYU, MINKI BAEK

**Affiliations:** 1 Department of Urology, Kyung Hee University Hospital at Gangdong, Kyung Hee University School of Medicine, Seoul, Republic of Korea; 2 Department of Urology, Samsung Medical Center, Sungkyunkwan University School of Medicine, Seoul, Republic of Korea; 3 Seoul Samsung Urology Clinic/Gynecology Health Care Center, Ulsan, Republic of Korea; 4 Department of Urology, Samsung Changwon Hospital, Sungkyunkwan University School of Medicine, Changwon, Republic of Korea

**Keywords:** vesico-ureteral reflux, dextranomer-hyaluronic acid copolymer, ultrasonography

## Abstract

Endoscopic treatment of vesicoureteral reflux (VUR) is feasible in pediatric patients. As endoscopic injection has a lower success rate than ureteral reimplantation, a postoperative voiding cystourethrogram (VCUG) is usually performed. The present study evaluated whether the presence of a nodule on noninvasive ultrasound could predict the resolution of VUR and replace invasive VCUG. Patients who received an injection of endoscopic bulking agent for VUR from January 2005 to December 2010 were evaluated retrospectively. It was evaluated whether a nodule, an echogenic mass lesion distinguished from the bladder at the ureteral orifice, was present on the ultrasound one month postoperatively. The success of the injection in the group with nodules was compared with that of the group without nodules by VCUG 3 months postoperatively. A total of 149 patients (220 ureters) met the inclusion criteria. The mean age at surgery was 3.5 years (range, 0.6–18 years). The overall success rate was 73.2%. A nodule was present in 152 cases (69.1%). The group with nodules had a higher success rate than the group without nodules (84.2%, 128/152 vs. 48.5%, 33/68, respectively; P<0.001). According to multivariate analysis, injection nodules were a predictive factor for the success of the endoscopic injection (odds ratio, 6.050; P<0.001). The failure rate increased with increasing injection volume. The sensitivity of sonographic injection nodules for predicting success was 79.5% and the specificity was 59.3%. To conclude, the presence of a postoperative nodule can predict the resolution of VUR.

## Introduction

Since it was first introduced by Matouschek (1) in 1981 and independently proposed by O’Donnell and Puri (2) in 1984, endoscopic injection has become popular for treating vesicoureteral reflux (VUR) due to its simplicity. The spread of endoscopic treatment accelerated with the introduction of dextranomer/hyaluronic acid (Dx/HA). Due to its advantages, including technical ease, minimal invasiveness, low complication rate and short hospital stay, endoscopic treatment is a viable alternative to open ureteral reimplantation. However, the rate of VUR resolution following injection treatment is lower than that following open ureteral reimplantation. In a review, it was reported that the overall success rate of injection ranged between 68 and 92% (3). Open ureteral reimplantation has a high success rate (94–99%) in correcting VUR, regardless of technique (4). Therefore, certain urologists insist that routine postoperative voiding cystourethrography (VCUG) is not necessary following open ureteral reimplantation (5–8). VCUG is an invasive procedure that requires a urethral catheter. However, the American Urologic Association (AUA) recommends a postoperative VCUG subsequent to injection treatment (9).

The AUA also recommends that following open surgical or endoscopic procedures for VUR, a renal ultrasound should be performed 1 month postoperatively to determine whether there are any obstructions (9). On the postoperative ultrasound, the echogenic injection material can often be visualized in the bladder. When an injection nodule is detected, it is hypothesized that the injection and maintenance of materials is successful. The present study evaluated whether the presence of an injection nodule on ultrasound can predict the resolution of VUR and replace invasive VCUG. The prognostic factors for success were also investigated.

## Materials and methods

### Patients

Patients who received an injection of endoscopic bulking agent for VUR at the Samsung Medical Centre (Seoul, Korea) between January 2005 and December 2010 were evaluated retrospectively. The research protocol was approved by the Samsung Medical Center Institutional Review Board. Patients with neurogenic bladder; posterior urethral valve; cloacal anomaly; previous open anti-refluxing surgery; ureteric abnormality such as duplication, diverticulum and ureterocele; insufficient medical records; or those who had not participated in an imaging study were excluded. The medical records were reviewed for each patient and the age at surgery, gender, affected side, VUR grade, injection material and treatment success were evaluated. VUR was graded according to the grading system of the International Reflux Study Committee (10).

### Injection procedure

All procedures were performed under general anesthesia with the patient in the lithotomy position. In the initial period, the subureteric transurethral technique (STING) was used. After 2007, the injection technique was changed to the hydrodistention-implantation technique (HIT). If the coaptation was insufficient following HIT, STING was also used. Polydimethylsiloxane (Macroplastique^®^; Uroplasty, Minnetonka, MN, USA) was injected until June 2006 and Dx/HA copolymer (Deflux^®^; Q-Med Scandinavia, Uppsala, Sweden) was injected thereafter.

### Postoperative examination

**Figure 1:**
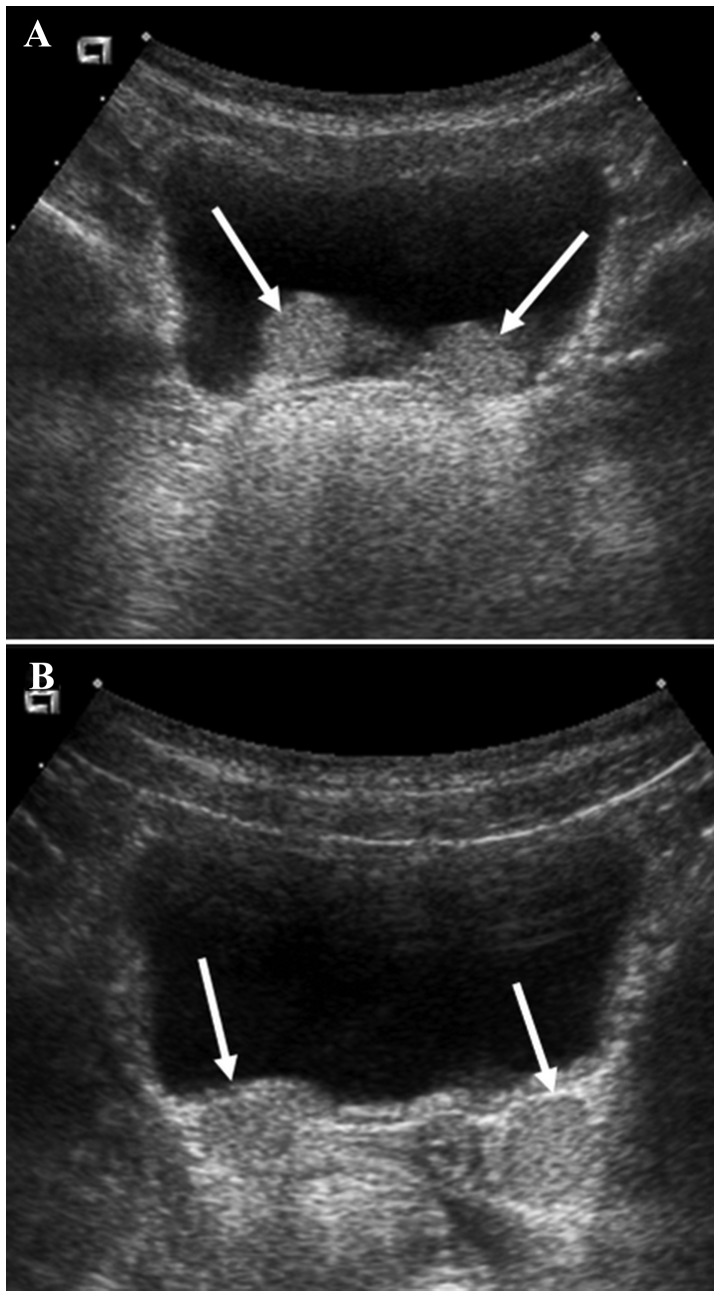
Ultrasonic images of an injection nodule. A nodule is defined as (A) a protruding mass lesion or (B) an echogenic mass, as indicated by the arrows.

Ultrasound was used to evaluate whether a nodule was present. To assess postoperative hydronephrosis, ultrasound was performed routinely in nearly all patients one month post-surgery. The ultrasound was performed on a full bladder by a pediatric radiologist. A nodule was defined as a protruding mass lesion (Fig. 1A) or echogenic mass (Fig. 1B) distinguished from the bladder at the ureteral orifice. At three months post-surgery, VCUG was performed to determine whether the VUR had been resolved. The injection treatment was considered successful if the VUR had disappeared on the postoperative VCUG at three months.

### Statistical analysis

The success rate in the group with nodules was compared with that in the group without nodules. To determine the value of an injection nodule as a diagnostic tool to predict VUR resolution, the sensitivity, specificity, positive predictive value (PPV), negative predictive value (NPV) and accuracy were calculated. The difference in treatment success according to non-numeric variables was assessed on univariate analysis with Pearson’s Chi-square test, Fisher’s exact test or the Cochran-Armitage test. Numeric variables were compared between treatment success and treatment failure by the Mann-Whitney test. A logistic regression analysis was conducted to evaluate the correlation between the variables and success. Variables are reported with 95% confidence intervals. Data were analyzed using PASW^®^ 18.0 (SPSS, Inc., Chicago, IL, USA) and P<0.05 was considered statistically significant.

## Results

### Patient data

Of 186 total patients, 149 patients (220 ureters) met the inclusion criteria. The mean age at surgery was 3.5 years (range, 0.6–18 years). Seventy-nine patients (53.0%) were male and 70 patients (47.0%) were female. Unilateral VUR was performed in 78 patients (52.3%) and bilateral VUR was performed in 71 patients (47.7%). There were 122 patients (81.9%) injected with Dx/HA and 27 patients (18.1%) injected with polydimethylsiloxane. The median injection volume was 1.13 ml (range, 0.1–4.2 ml).

### Univariate analysis of success rate

Among the 220 ureters, 161 ureters (73.2%) exhibited a complete resolution of VUR on postoperative VCUG. The success rates of VUR were 82.6% (19/23) for Grade I, 89.3% (25/28) for Grade II, 72.0% (67/93) for Grade III, 66.7% (44/66) for Grade IV and 60.0% (6/10) for Grade V. As the VUR grade increased, the success rate tended to decrease (P=0.018).

Injection nodules were present in 152 ureters (69.1%). Of these, VUR resolved in 128 ureters (84.2%). The group with injection nodules had a higher success rate than the group without injection nodules (84.2 vs. 48.5%, respectively; P<0.001). There was a positive correlation between the presence of the injection mounds and VUR resolution. On univariate analysis, gender, laterality and injection material did not significantly influence the success rate (Table I). The mean injection volume in the group with VUR resolution, however, was significantly smaller than that in the group with persistent VUR (1.05 vs. 1.38 ml, respectively; P=0.001).

### Multivariate analysis of success rate

On multivariate analysis, injection nodules were predictive of endoscopic injection success (odds ratio, 6.050; P<0.001). The failure rate increased with increasing injection volume (odds ratio, 0.428; P=0.004; Table II). Sonographic injection nodules had 79.5% sensitivity, 59.3% specificity, 84.2% positive predictive value, 51.5% negative predictive value and 74.1% accuracy as a diagnostic tool for success rate. These values increased slightly with increasing VUR grade (Table III).

## Discussion

As endoscopic injection has a lower success rate than open surgical reimplantation, there have been numerous efforts to identify good candidates for endoscopic injection treatment and predictive factors for success (11–15). The prognostic factors can be divided into preoperative factors and treatment-associated factors. The preoperative factors are patient-dependent factors, such as VUR grade, anatomic bladder and ureteral abnormalities and dysfunctional voiding; preoperative VUR grade is a chief prognostic factor. Higher VUR grades are associated with lower success rates (13,14,16).

Although there are discrepancies among studies, the known treatment-associated factors are surgeon-dependent factors such as surgeon experience, injection technique, mound morphology and location, and injective volume. These factors are associated with the outcome of the endoscopic injection treatment. The goal of endoscopic injection treatment is to create a subureteral mound that is able to elevate and coapt the ureteral orifice. A satisfactory mound is the most important factor in the success of Dx/HA injection, following adjustment for other factors such as VUR grade and the volume injected (13).

Whether the presence of an injection nodule on a postoperative ultrasound can predict the resolution of VUR has remained uncertain until now. Few studies have investigated the association between sonographic injection nodules and the success of endoscopic injection (17–19). A polydimethylsiloxane implant was identifiable in 84% of ultrasounds in one study and 86% of these had corrected VUR on postoperative VCUG (19). In addition, ultrasound had a sensitivity of 89% and specificity of 86% for VUR correction. Another study, however, reported no correlation between the presence of a Dx/HA nodule and the resolution of VUR on VCUG (17). In the present study, patients with an injection nodule had a higher success rate than patients without an injection nodule. On multivariate analysis, injection nodules were predictive factors for the success of endoscopic injection. Contrary to the results of Ellsworth *et al* (17), the sensitivity and specificity were relatively low in the present study. Therefore, it is concluded that the presence of an injection nodule on postoperative ultrasound cannot replace VCUG.

In the present study, 24 ureters (10.9% of the total cases) with injection mounds had sustained VUR following endoscopic injection, which may have several explanations. The injection material could have been in the wrong position. When performing a second injection for failed cases, implants were observed in improper locations. Another explanation may be insufficient coaptation of the ureter.

In 68 cases (30.9% of the total cases), injection mounds were not detected. These injection mounds were likely to have been missed by the radiologist either because they were too small or because they were absent. An insufficient volume could be due to too little material being injected or the injection material being spilled. If the bladder mucosa overlying the injection material was eroded, the material may have been expelled during voiding.

The VUR resolved in certain cases without sonographic injection nodules. This finding may be explained by tissues reacting with the injection materials. Hydrolysis of dextranomer microspheres reduces the volume of the injected materials, but endogenous collagen production between the microspheres results in tissue augmentation (20).

In 2002, Oswald *et al* compared a single endoscopic injection of polydimethylsiloxane with Dx/HA for the treatment of VUR in children (21). VUR was corrected in 86.2% of the children injected with polydimethylsiloxane and in 71.4% of the children injected with Dx/HA at the three-month follow-up visit. No postoperative complications were observed in either group. The success rates in the current study, which were 79.5% for the children injected with polydimethylsiloxane and 71.8% for the children injected with Dx/HA, are consistent with the data from the previous study. Also, no significant differences were identified between the two groups in the present study (P=0.327).

In the present study, increased injection volumes were identified to be associated with injection failure. This is comparable to the findings of a previous report (13) and may be due to difficulty in creating a proper mound with larger injection volumes. If the ureteral orifice is wide or the distal ureter is dilated, a greater volume might be necessary to coapt the ureteral orifice. These cases are also more likely to fail than low grade VURs. By contrast, smaller volumes indicated success in creating a mound and an increased likelihood of reflux resolution.

In conclusion, the presence of a postoperative injection nodule is able to predict resolution of VUR. However, the sensitivity and specificity are relatively low. If a postoperative injection mound is present on ultrasound examination, the child’s parents should be informed of the high probability of success prior to performing VCUG.

## Figures and Tables

**Table I:** Success rate according to each variable and univariate analysis between variables and success.

Variables	No. of ureters, n (%)	Success rate, n (%)	P-value
Nodule			<0.001
Present	152 (69.1)	128 (84.2)	
Absent	68 (30.9)	33 (48.5)	
Gender			0.378
Male	116 (52.7)	82 (70.7)	
Female	104 (47.3)	79 (76.0)	
Laterality			0.184
Right	102 (46.4)	79 (77.5)	
Left	118 (53.6)	82 (66.5)	
Injection material			0.327
Dx/HA	181 (82.3)	130 (71.8)	
Polydimethylsiloxane	39 (17.7)	31 (79.5)	
Grade			0.018^a^
I	23 (10.5)	19 (82.6)	
II	28 (12.7)	25 (89.3)	
III	93 (42.3)	67 (72.0)	
IV	66 (30.0)	44 (66.7)	
V	10 (4.5)	6 (60.0)	

a Calculated by Cochran-Armitage test.

Dx/HA, dextranomer/hyaluronic acid copolymer.

**Table II:** Multivariate analysis between variables and success.

Risk factors	Odds ratio	95% confidence interval	P-value
Nodule	6.050	2.998–12.209	<0.001
Age	1.079	0.969–1.201	0.164
Gender (female)	1.051	0.519–2.131	0.890
Laterality (left)	0.671	0.334–1.350	0.264
Injection material (polydimethylsiloxane)	1.079	0.430–3.170	0.761
Injection volume	0.428	0.240–0.761	0.004
Grade			
I	1	–	–
II	1.555	0.272–8.906	0.620
III	0.541	0.146–2.004	0.358
IV	0.569	0.144–2.254	0.422
V	0.694	0.107–4.515	0.702

**Table III:** Diagnostic values of sonographic injection nodules (%).

Grade	Sensitivity	Specificity	PPV	NPV	Accuracy
I	68.4	50.0	86.7	25.0	65.2
II	80.0	66.7	95.2	28.6	78.6
III	80.6	50.0	80.6	50.0	72.0
IV	81.8	63.6	81.8	63.6	75.8
V	83.3	100.0	100.0	90.0	90.0
Total	79.5	59.3	84.2	51.5	74.1

PPV, positive predictive value; NPV, negative predictive value.
